# Analysis of Population Structure in Hungarian Coldblood Horses Based on Pedigree Information

**DOI:** 10.3390/ani15101406

**Published:** 2025-05-13

**Authors:** Brigitta Barsi, János Oláh, János Posta

**Affiliations:** 1 Department of Animal Husbandry, Institute of Animal Science, Biotechnology and Nature Conservation, Faculty of Agricultural and Food Sciences and Environmental Management, University of Debrecen, H-4032 Debrecen, Hungary; barsibrigi@mailbox.unideb.hu; 2 Doctoral School of Animal Science, University of Debrecen, H-4032 Debrecen, Hungary; 3 Farm and Regional Research Institute of Debrecen, University of Debrecen, H-4032 Debrecen, Hungary; olahja@agr.unideb.hu

**Keywords:** Hungarian Coldblood breed, pedigree analysis, pedigree quality, gene origin, inbreeding

## Abstract

The Hungarian Coldblood horse was developed in the 1920s by crossing local mares with draft horses imported from Belgium and France, and was approved as an official horse breed in 1954. The aim of the study was to analyze the quality of the pedigree, generation interval, gene origin, and inbreeding. The pedigree information was received from the Hungarian Coldblood Horse Breeding Association. The studbook data of the registered animals up to 2023 were evaluated. Two reference populations were chosen: horses having offspring in 1989 and 2023. The final database contained 21,699 horses. Pedigree data of the total population and the actual breeding stock were analyzed using Endog 4.8. and Grain 2.2. software. The complete generation equivalent was 4.64 and 7.72, whereas the average maximum generations was 7.90 and 13.06 for the total and reference population, respectively. There were significant differences between the parent–offspring pathways of generation intervals. In the total stock, the first 10 individuals are present in 26.71%, while in the actual breeding stock, they are present in 37.84%. The average Wright’s inbreeding coefficient was 1.13% and 2.35% in the total population and the actual breeding stock. Kalinowski’s new decomposition of inbreeding showed that inbreeding does not originate from the past; inbreeding is stronger in more recent generations. There was a reasonable bottleneck effect. A more careful mating design might be needed to avoid inbreeding in the future.

## Simple Summary

Maintaining genetic variability is the most important purpose of animal conservation programs. The Hungarian Coldblood horse is an indigenous breed which is descended from horses traditionally used by peasants. In the last century, the role of horses has changed, and they have been replaced by technical innovations and motorization, thus reducing the population size and genetic diversity. The aim of this study was to provide information on the current breeding stock and to support breeders’ associations in their gene conservation work for this endangered local breed. The pedigree quality, generation intervals, probability of gene origin, and inbreeding were assessed. We found that the breed had a large bottleneck effect during its breeding history. The level of inbreeding was measured using different methods, such as Ballou’s, Wright’s, and Kalinowski’s coefficients.

## 1. Introduction

Heavier-type horses from Western Europe probably appeared in Hungary for the first time during the reign of the Árpád dynasty and later as companions of various military campaigns and royal delegations, but their significant spread can be attributed only to the 1880s [1].

The carting trade that began in the counties of Zala, Vas, and Sopron affected Vienna and the nearby Austrian provinces. This work required strong, unpretentious, and easy-to-handle horses. The western Hungarian carters were happy to import the Noriker and Pinzgau horses or to mate their warm-blooded mares with heavier-type stallions [2]. Due to the irregular use and crossbreeding with different breeds and types, the population was a rather mixed and heterogeneous [3].

In 1922, the registration of cold-blooded horses began in Hungary. The imports from Belgium continued in the 1930s, resulting in a sub-population within the Hungarian cold-blood horse quite similar to the Belgian–Ardennes type, mainly in Baranya County [1]. Between 1948 and 1949, 59 Belgian–Ardennes stallions from Belgium as well as 17 French-Ardennes cold-blooded stallions from France were imported and used in public breeding. Planned crossbreeding was carried out with these stallions [4]. The genotype and phenotype of the Hungarian Coldblood horse population changed under the influence of imported stallions, resulting in the creation of an independent, new breed. This transformed breed was registered as the Hungarian Coldblood in 1954 [3]. The development of agriculture also changed the order of exploitation of the cold-blooded horse in the 1980s. More French Percherons and Belgian imports were introduced into breeding to increase the body weight of the Hungarian breed. Unfortunately, this also affected the genetic structure of the breed [3].

**Figure 1:**
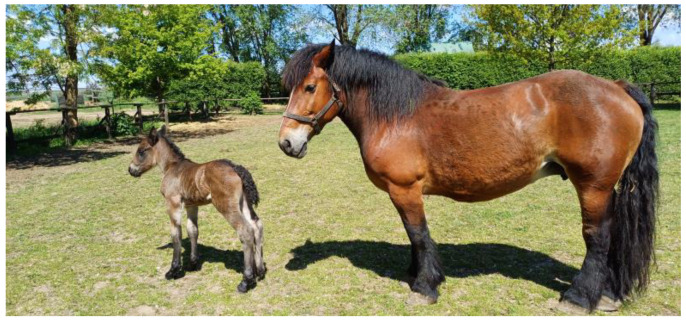
Hungarian Coldblood breeding mare along with her foal. Photo by János Oláh.

Hungarian Coldbloods are more solidly built, less demanding, and more agile than Western European Coldbloods. Hungarian Coldblood horses have a calm temperament and mature quickly (Figure 1). They are excellent workers, with a good temperament, and are easy to handle. Traditionally, they were reliable and friendly workers for farmers, who learned quickly and did not require special animal husbandry skills. Because of this, it has recently become popular as a leisure animal [5]. Nowadays, this medium-sized draught horse breed has an open studbook. The height at withers ranges between 150 and 175 cm, whereas the girth circumference is at least 120% of the withers height, but generally falls between 125% and 130%. From the age of three, the minimum cannon bone circumference is 23 cm for mares and 24 cm for stallions.

32/2004. (IV. 19) [6] declared the Hungarian cold-blooded breed a national treasure. Maintaining genetic variability is the most important task of the gene conservation program. An effective gene conservation program requires knowledge of the genetic diversity of the population. The genetic structure and variability of a population can be assessed using pedigree data, which offers detailed information regarding the ancestors and relatives of the horses within the population [7].

In this study, our objectives were to gather information on the current breeding population and to support the breeder association in their gene conservation program. Assessing genetic variability within a population can yield valuable insights into the breed’s history and current status, which are essential for designing an effective breeding program.

## 2. Materials and Methods

### 2.1. Pedigree Analysis

**Figure 2:**
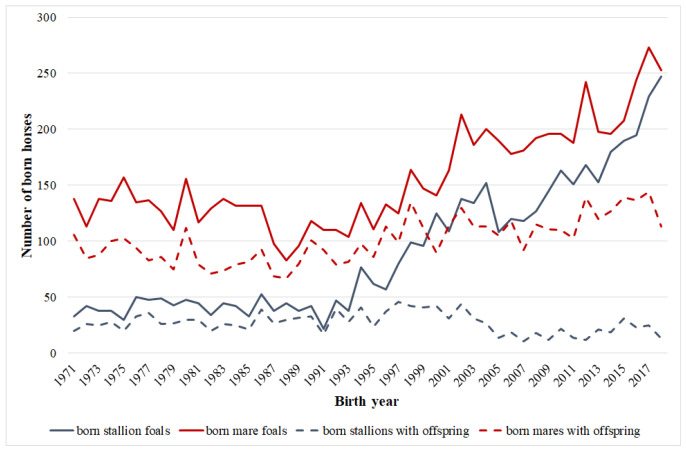
Total number and number of selected foals for breeding across time in the Hungarian Coldblood population.

The pedigree information was received from the Hungarian Coldblood Horse Breeding Association. Pedigree data from 1805 to 2023 was analyzed. There were the pedigree data of 21,699 animals in the developed database. There were two reference populations during the analyses. The first included horses having offspring at the foundation year (1989) of the breeder association, whereas the second was the active (horses having offspring) breeding population from 2023. These populations are characterized in Table 1, whereas population size changes in the breed could be followed through a number of born foals (Figure 2). The reference population is the individuals of the entire population born in a given period of time [8].

The developed database was set up in Microsoft Access 2016. It contained the name of the individual, the name of the sire, the name of the dam, the birth date, and the sex of the horse. The database was checked for loops in the pedigree as well as for bisexual (animal appears as stallion and mare in the database) and duplicate horses, but no records had to be removed.

The pedigree analysis was conducted using Endog v4.8 software (Madrid, Spain) [9] and POPREP v2.0 (Neustadt, Germany) software [10]. Prior to the analysis, the database was verified using the Pedigree Viewer v6.5 software (Armidale, Australia) [11]. Various inbreeding coefficients were estimated using Grain v2.2 software (Wien, Austria) [12].

### 2.2. Pedigree Completeness

This parameter determines the pedigree quality and usability. When we use pedigree analysis, we need a minimum of 3–4 fully known generations [13]. Pedigree completeness can change the inbreeding coefficient value; the more unknown ancestors, the more underestimated the coefficient [14,15].

It can be interpreted in several ways:

- Maximum number of generations (GenMax)—the number of generations separating the individual from its furthest ancestor [7];
- Number of complete generations (GenCom)—the furthest generation where all ancestors of the individual are known [7];
- Equivalent complete generations (GenEqu)—defined as the sum of the proportion of known ancestors over all generations traced. It could be computed as the sum over all known ancestors of the terms computed as the sum of (1/2)^n^, where n is the number of generations separating the individual from each known ancestor [7].

### 2.3. Generation Interval

The generation interval was defined as the average age of parents when their offspring is born [16]. Generation intervals were estimated on four different pathways separately in this study: sire-to-daughter, sire-to-son, dam-to-daughter, and dam-to-son ways [17]. The four pathways were compared pairwise (*p* < 0.05) for each using an independent samples *t*-test using the formula:

t=X1¯−X2¯sp1n1+1n2, where sp2=(n1−1)s12+(n2−1)s22n1+n2−2.

### 2.4. Probability of Gene Origin

- Number of founders (N_f_)—the number of animals with unknown parents [18];
- Number of ancestors (N_a_)—the minimum number of individuals in the pedigree, which explains the total genetic variability in the population [18];
- Effective number of founders (f_e_)—the number of animals that, if mated randomly, would produce the same amount of genetic variation as the study population [15];
- Effective number of ancestors (f_a_)—the marginal contributions of ancestors that would be expected to produce the same genetic diversity as in the population under study [15].

### 2.5. Inbreeding Coefficient and Average Relatedness

The inbreeding coefficient (F) is the degree of relationship between two individuals who share common ancestors, and it was estimated using the formula proposed by Wright [19].

- Wright method (F_Wright)—the probability that the two alleles at any locus in an individual are identical by descent [19];
- Ballou method (F_Ballou)—the probability that an individual inherits an allele which has undergone inbreeding in the past at least once [20];
- Kalinowski method (F_Kal) and Kalinowski new method (F_Kal_new)—classical inbreeding coefficient is split into two parts, alleles which had undergone inbreeding in the past (‘old’, i.e., F_Kal) and alleles identical by descent for the first time (‘new’ inbreeding coefficient, F_Kal_new). Thus, F_Kal represents the part of the genome where alleles are currently in identical by descent status and have also been identical by descent in the ancestor of the animal at least once [12,21];
- Average relatedness (AR)—the probability that an allele randomly selected from the entire population belongs to the individual [22].

## 3. Results

### 3.1. Pedigree Completeness

**Figure 3:**
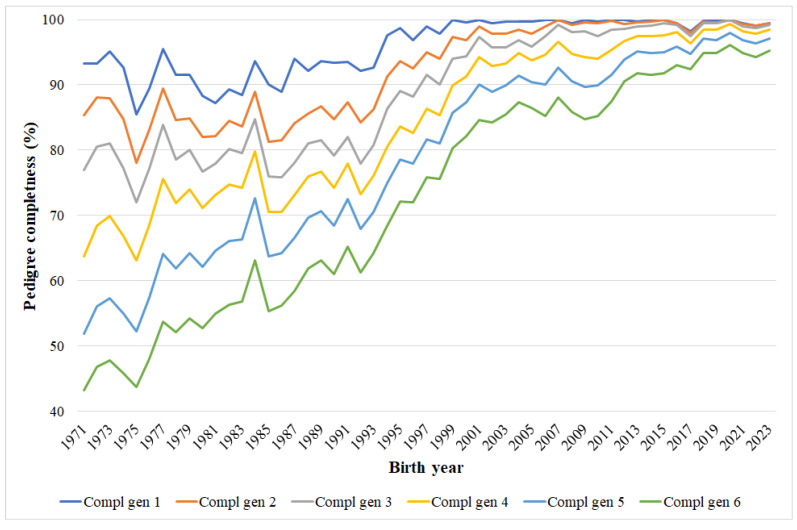
Changes in pedigree completeness in the Hungarian Coldblood population.

The estimated pedigree completeness values for Hungarian Coldblood horses are shown in Table 2 and illustrated in Figure 3.

The average value of the maximum number of generations was 7.90 in the total population. There were 10,411 individuals that had at least one known ancestor in their ‘pedigrees’ 10th or later generations out of the 21,699 horses. The deepest pedigree was 27 generations long for the mare named Linda. The number of complete generations was 2.80. At least five generations of pedigree information were completely known for 5388 horses, which was 24.38% of the total population. The equivalent complete generation was 4.64. The highest value was 10.16, which belonged to the mare named Anikó.

The pedigree completeness values were quite low for the breeding stock in 1989, but quickly increased after the foundation of the breeding organization (Figure 3). In the actual breeding stock, the mean number of maximum number of generations was 13.06. Among the 1123 individuals, 812 animals can be traced back 13 or more generations. There were four horses in the current breeding stock which had known ancestors up to the 16th generation. The average number of complete generations was 4.60. At least five complete generations were known for 56.19% of the actual breeding stock. The pedigree was fully known back to seven generations for five individuals. The mean number of complete generations equivalent was 7.72. The mare named Orgona had the highest value, which was 9.71.

### 3.2. Generation Interval

The estimated generation intervals are presented in Table 3. The four pathways were compared pairwise, using an independent samples *t*-test, revealing significant differences among all pathways. The longest generation interval was observed in the sire-to-son pathway, with a duration of 10.14 years, while the shortest was recorded in the dam-to-daughter pathway, estimating 8.49 years. The average generation interval was calculated to be 9.17 years.

### 3.3. Probability of Gene Origin Based on Parameters

Table 4 shows the estimated genetic variability for the total and the reference populations.

The 21,699 Hungarian Coldblood horses registered in the total population originated from the genetic contribution of 3996 founders (N_f_) and 3169 ancestors (N_a_). The effective number of founders (f_e_) was 278, and the effective number of ancestors (f_a_) was 95.

The horses in the actual breeding stock were derived from the contributions of 1501 founders and 415 ancestors. The effective number of founders was 183, and the effective number of ancestors was 49. The f_a_/f_e_ ratio was 0.342 in the total population and 0.268 in the actual breeding stock, respectively. The obtained ratios support the occurrence of the bottleneck effect in the reference as well as the total population.

Table 5 provides information on the concentration of genetic variability in the analyzed populations. In the total population, 44 horses accounted for 50% of the total genetic variability, whereas in the actual breeding stock, this was achieved by only 17 horses. The entirety of genetic variability was represented by 3169 out of 21,699 horses in the total population and by 415 out of 1123 horses in the actual breeding stock. The breeding stock in 1989 was more heterogeneous compared to the actual breeding stock.

Table 6 presents the contribution of the ten most influential ancestors to the genetic variability. These ancestors accounted for 26.71% of the genetic diversity in the total population and 37.84% of the actual breeding stock. The most important ancestors covered 4.59% and 5.68% of the genetic diversity.

### 3.4. Inbreeding Level and Average Relatedness

Table 7 gives an overview of the homozygosity of the Hungarian Coldblood horse. The inbreeding was calculated in different ways.

The average inbreeding coefficient of the total population was 1.13%, that of the actual breeding stock was higher—2.35%. The proportion of inbreeding was 49.89% and 91.36% in the total population and the actual breeding stock. In the actual breeding stock, 101 individuals had an inbreeding coefficient over 5%.

The estimated ancestral inbreeding coefficients provide insight into whether inbreeding occurred in the past or mainly in the present generations. The results indicate that the recent inbreeding (F_Kal_new) result was higher than ancestral inbreeding (F_Kal) in both the total and reference breeding populations. This suggests that the observed homozygosity is primarily a recent phenomenon rather than a legacy of past generations.

**Figure 4:**
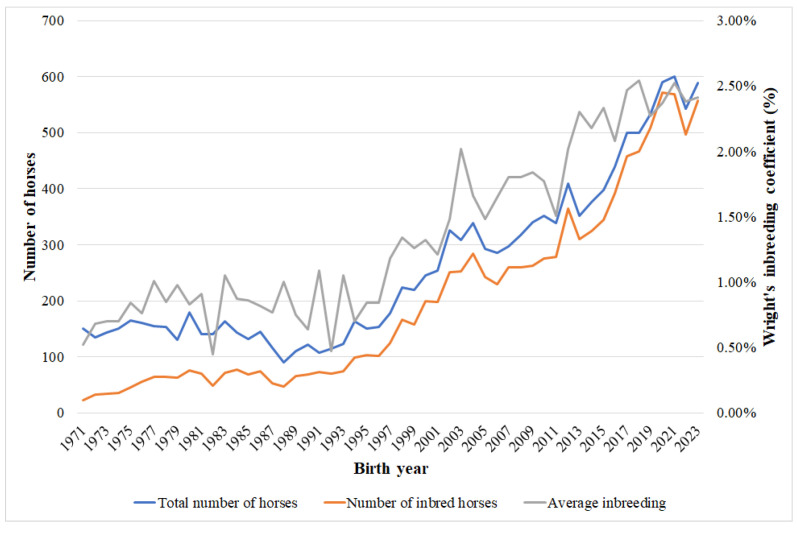
Changes in inbred horses and inbreeding in the Hungarian Coldblood population.

The annual changes in inbred horses and average inbreeding are illustrated in Figure 4. The increase in inbred animals aligns with the increase in the population. The annual increase in inbreeding might be the result of the lack of imported animals in recent years, as well as the deeper pedigree information.

The probability of an allele being homozygous in previous generations was nearly 2.5% and 5% in the total population and the actual breeding stock.

Average relatedness in the total population was 1.32%, which is lower than the 2.3% of the actual breeding stock. The AR values exceeded half of Wright’s coefficient, indicating that mating occurred between related individuals.

## 4. Discussion

### 4.1. Pedigree Completeness

A more comprehensive pedigree enables more precise estimations by reducing the number of unknown ancestors. The accuracy of inbreeding level estimation strongly depends on the depth and completeness of the pedigree data.

In the total population, the GenCom value was 2.8. This is higher than in Serbian and Bosnian Lipizzan horses (2.03 and 2.31) [23]. The average number of complete generations was 4.60 for the actual breeding stock, which is lower than it was estimated (4.95) for the Furioso–North Star and Nonius breeds [24]. In the Murgese horse, this value was quite similar (4.52) [25] to our findings. In the literature, the lowest value was 0.49 in Pantaneiro breed [26] and the highest value was 6.9 in Polish Arabian horses [27].

The maximum number of generations of Serbian Lipizzan horses was 12.84 in the actual breeding stock [23]. This value was the closest to our actual breeding stock (13.06). The shortest average pedigree line is found in Pantaneiro horses with a generation value of 0.95 [26]. This generation value is notably lower than the 36.56 generation value in Furioso–North Star horses [24].

The average equivalent complete generations in the actual breeding stock was in agreement with the estimation for the Brazilian Quarter Horse population (6.24) [28] and the Murgese horse population (6.98) [25]. In the Turkish Arab horse, GenEqu was quite similar (7.8) [29]. In the total population of Sardinian Anglo-Arab, this value was 4.00 [30]. In articles, the average GenEqu values were between 0.67 (Pantaneiro) [26] and 51.10 (Sztumski) [31]. Compared to the values of the total population and the breeding stock in 1989, the values of the actual breeding stock are almost double. This strengthens the proper administrative work of the breeding association and supports a certain hypothesis that the pedigree of the actual breeding stock was longer and better known.

The increasing trend of the pedigree completeness values across time clearly shows the breeding history. The breed still has an open stud book, but after the registration of the breed, animals with unknown parents were not allowed to be used.

### 4.2. Generation Interval

The generation interval has a significant impact on the genetic development of a population, it is usually longer for horses compared to other livestock species. In horse breeding, the average and optimal generation interval is considered to be a period between 8 and 12 years, during which the most efficient genetic development occurs. The generation interval depends on the sex of the animal, while males and females may be at different ages when their selected offspring are born. Therefore, it is important to analyze the generation interval for the population as a whole and for males and females separately [32]. As expected, sire pathways were longer than dam pathways. In the Franches–Montagnes breed, the generation interval between dam-to-son (8.6) [33] was close to our value. This may be a result of the selection method used in breeding stallions. From this, it can be concluded that breeders are more careful when selecting stallions than when classifying mares as breeding animals. The use of extended generation intervals is favorable for gene conservation. This breeding strategy results in slower genetic progress but can help to mitigate gene loss. Additionally, it may contribute to maintaining genetic diversity, which is a crucial consideration in gene conservation efforts. The observed average generation interval was comparable to previously reported values for other breeds, including 9 years in Campolina horses [34], 8.6 in Dutch harness horses [35], and 9.6 in Cleveland Bay horses [36]. The lowest generation interval (7.24 years) was described for the Sokólski breed [31]. The longest generation interval, 12.27 years, was described for the Shagya Arabian breed [37].

### 4.3. Probability of Gene Origin Based on Parameters

The calculated f_e_ value for the actual breeding stock was between the values of the Shagya (160) and Pantaneiro (312) breeds [26,37]. The f_e_ value in the Sardinian Anglo–Arab breed (287) [30] was quite similar to that in the total population of Hungarian Coldblood. The reported effective number of founders covers a large interval in the literature. The fewest effective number of founders is found in the Mangalarga breed (36) [38], while the most effective number of founders can be counted in the entire Brazilian Quarter Horse population (1045) [28].

The f_a_ value of the actual breeding stock was slightly lower than the value of 51 for the Italian Heavy Draught Horse [39]. The lowest effective number of ancestors (9) was found in Cleveland Bay [36]. The highest figure (297) was found in the Brazilian Pantaneiro breed [26].

The f_a_/f_e_ ratios showed a bottleneck effect for each population. It can be observed that the ratio pair value is lower in the reference stock, so the bottleneck effect is stronger in this one. The bottleneck effect affects the decrease in genetic variability. The ratio of the effective number of ancestors to the effective number of founders in the actual breeding stock was 0.268. The value is between the values of the Gidran (0.24) and Furioso–North Star breeds (0.43) [23]. For the reference population of the Murgese horse, this ratio was twice as high (0.50) [25]. The minimum value (0.23) was reported for the Nonius breed [24]. The maximum value (2.74) was calculated for the Hungarian English Thoroughbred population [18].

The breeding stock in 1989 was quite small but diverse, as f_e_ and f_a_ values were quite similar to the values of the total population. Probably due to the open studbook, N_f_ and N_a_ values for the actual breeding stock are higher compared to the breeding stock in 1989. The differences between the total population and the actual breeding stock values for the same genetic diversity ratios were substantial, particularly at the values of f_a_80, f_a_90, and f_a_100. This indicates gene loss, as the genetic coverage is attributed to fewer ancestors. While decades of selective breeding and associated methodologies may partially account for this trend, the real reason is much more the loss of the original sire lines and the disproportionate utilization of remaining breeding animals. In American Shire horses, only 39 ancestors explained 80% of the gene pool [40], which is quite smaller compared to our findings.

The total value of the first 3 individuals is 12.41% of the total population, in reference to 16.07%, which is higher by almost 4%. The aggregate value of the first 10 individuals in the total population is 26.71%, while in the reference herd it is 37.84%; here, the reduction is more than 11%. The genetic contribution of the first ten founders in the Hungarian Thoroughbred horse population, which originally consisted of 1062 founders, was 34.86% [18].

### 4.4. Inbreeding Level and Average Relatedness

The inbreeding coefficient plays a crucial role in maintaining the genetic diversity of a breed. Inbreeding is feared when breeding breeds that are in gene conservation, because it means an increase in homozygosity, which reduces genetic diversity. For the population, the Wright inbreeding coefficient ranged between the values reported for the Italian Heavy Draught Horse (2.28%) [39], American Shire (2.4%) [40], and Campolina (2.45%) [34] breeds. The lowest value (0.04%) was described for the Pantaneiro breed [26]. The highest value (13%) was calculated for Kladruber horses [41]. Compared to other indigenous Hungarian horse breeds, this inbreeding value is considered very favorable. This is due to the fact that the breed is a relatively young breed, and its studbook is open.

Kalinowski’s and Kalinowski’s new inbreeding coefficients have not been extensively studied in horse breeding. According to its definition, F_Kal accounts for alleles that have become homozygous due to common ancestry in the past, thereby considering only the ancestral inbreeding. Consequently, F_Kal remains zero for an individual when its F_Wright is zero. The results indicated that recent inbreeding (F_Kal_new) was higher than ancestral inbreeding (F_Kal) in both the total and reference breeding population. This suggests that the observed homozygosity is of recent origin rather than originating from the past. This might be the result that, up to the foundation of the breeding organization lot of mares were entered into the breeding with very short pedigree information. A possible other explanation is that other cold-blooded breeds are no longer used in breeding. The values of the actual breeding stock are double those of the total population. In small, native, endangered breeds, mating of related individuals is often unavoidable; however, the birth of individuals resulting from close related matings (parent–offspring, grandparent–grandchild, sibling/half-sibling matings) should be avoided, and the planning of these matings requires great expertise. Such mating results in a decrease in diversity; therefore, in the case of breeds under a gene conservation program, its mitigation and avoidance are a task for breeders and breeding associations. Certain arguments (e.g.,: more uniform inheritance, recording of characteristics, pedigree characteristics) may justify the birth of such offspring, but special attention should be paid to these individuals in their further breeding.

The average relatedness (AR) values were higher than half of Wright’s inbreeding coefficient, indicating that mating between related individuals occurred in the breed. The AR value for the total population value was slightly different from the value of the total population of Italian Heavy Draught Horse (1.39%) [39], while AR for the actual breeding stock value exceeded the value for Campolina horses (2.0%) [34]. The average relatedness values ranged from 0.13% to 21%, with the lowest reported for the Pantaneiro horse breed [26] and the highest for the Kladruber horse breed [41].

## 5. Conclusions

The average value of equivalent complete generations provides a reliable basis for estimating pedigree-based population genetic parameters in future works in the actual breeding stock. The results can serve as a guideline for determining target matings to reduce the incidence of inbreeding for the next offspring generations. Since inbreeding is higher in new generations, and there was a reasonable bottleneck effect in the population, more attention is needed when determining mating plans in the future. The application of SNP-chip analyses could strengthen our results, so it might be favorable.

## Figures and Tables

**Table 1:** Numerical description of the Hungarian Coldblood populations.

Parameter	Total Population	Actual Breeding Stock	Breeding Stock in 1989
Total number of horses	21,699	1123	182
Number of stallions	8269	216	70
Number of mares	13,430	907	112

**Table 2:** Pedigree completeness values of Hungarian Coldblood populations.

Parameter	Total Population	Breeding Stock in 1989	Actual Breeding Stock
N	21,699	182	1123
GenMax	7.90	5.31	13.06
GenCom	2.80	2.18	4.60
GenEqu	4.64	3.34	7.72

N: number of horses; GenMax: maximum number of generations; GenCom: number of full generations traced; GenEqu: equivalent complete generations.

**Table 3:** Generation intervals in the Hungarian Coldblood populations.

Pathways	Numbers	Generation Interval (Years)	Deviation
Sire-to-son	1715	10.14 ^a^	4.81
Sire-to-daughter	5562	9.51 ^b^	4.45
Dam-to-son	1455	8.93 ^c^	3.82
Dam-to-daughter	4719	8.49 ^d^	3.90
Average	13,451	9.17	4.29

^a,b,c,d^: Different superscript letters show significant differences (*p* < 0.05).

**Table 4:** Ancestors and founders of the Hungarian Coldblood populations.

Parameter	Total Population	Breeding Stock in 1989	Actual Breeding Stock
N_f_	3996	708	1501
N_a_	3169	205	415
f_e_	278	238	183
f_a_	95	99	49
f_a_/f_e_	0.342	0.416	0.268

N_f_: number of founders; N_a_: number of ancestors; f_e_: effective number of founders; f_a_: effective number of ancestors.

**Table 5:** Concentration of genetic variability for Hungarian Coldblood populations.

Parameter	Total Population	Breeding Stock in 1989	Actual Breeding Stock
N_a_50	44	54	17
N_a_60	83	72	27
N_a_70	168	91	42
N_a_80	414	114	72
N_a_90	1161	144	132
N_a_100	3169	205	415

N_a_50; N_a_60; …; N_a_100: number of ancestors contributing for 50%; 60%; …; 100% of the genetic variability in the population.

**Table 6:** Ancestral contribution to the genetic variability for Hungarian Coldblood populations (%).

Parameter	Total Population	Breeding Stock in 1989	Actual Breeding Stock
first ancestor	4.59	4.18	5.68
second ancestor	4.07	3.93	5.22
third ancestor	3.75	2.94	5.17
first 3 ancestors	12.41	11.04	16.07
first 10 ancestors	26.71	22.57	37.84

**Table 7:** Homozygosity coefficients of Hungarian Coldblood population.

Parameter	Total Population	Breeding Stock in 1989	Actual Breeding Stock
F_Wright (%)	1.13	0.77	2.35
F_Kal (%)	0.18	0.03	0.39
F_Kal_new (%)	0.95	0.74	1.96
F_Ballou (%)	2.47	0.73	4.95
AR (%)	1.32	1.11	2.30

AR: average relatedness; F_Wright: inbreeding coefficient; F_Ballou: Ballou’s formula for ancestral inbreeding; F_Kal: identical alleles were inbred in the past; F_Kal_new: identical alleles were inbred in recent generations.

## Data Availability

The data presented in this study are available on request from the corresponding author. The data are not publicly available due to privacy restrictions.
